# The Role of Cerebrospinal Fluid Biomarkers in Dementia and Other Related Neurodegenerative Disorders

**DOI:** 10.3390/brainsci12050627

**Published:** 2022-05-11

**Authors:** George P. Paraskevas

**Affiliations:** 1 2nd Department of Neurology, School of Medicine, National and Kapodistrian University of Athens, “Attikon” General University Hospital, 12462 Athens, Greece; geoprskvs44@gmail.com; Tel.: +30-2105832466; 2 1st Department of Neurology, School of Medicine, Neurochemistry and Biological Markers Unit, “Eginition” Hospital, 11528 Athens, Greece

Over the course of the last 20 years, cerebrospinal fluid (CSF) biomarkers for Alzheimer’s disease (AD), including amyloid beta peptide with 42 amino acids (Aβ_42_), total tau protein (τ_T_), and tau protein phosphorylated at a threonine residue at position 181 (τ_P-181_), have become a useful tool for the recognition and diagnosis of AD, even in early or atypical clinical presentations and in the presymptomatic stage of the disease [1]. However, there are still arguments concerning the definition of the CSF biomarker AD profile, while in the differential diagnosis of AD, forming other neurodegenerative disorders or psychiatric disorders, additional biomarkers may be necessary, including as α-synuclein (α-syn), neurofilament light chain (NFL) and TAR DNA-binding protein 43 (TDP-43). Furthermore, blood-based biomarkers may prove to be a useful adjunct or alternative to CSF biomarkers in the screening, diagnostic workup, and follow-up of dementia patients.

This Special Issue of *Brain Sci.* entitled “Cerebrospinal Fluid Biomarkers in Dementia Disorders” includes seven articles dealing with the role of CSF (but also plasma) biomarkers in the understanding of the biochemical mechanisms and the (differential) diagnosis of dementing disorders.

McGrowder et al. [2] reviewed the current status of established (core) CSF biomarkers of AD and the possible role of emerging biomarkers, including not only α-syn and TDP-43, but also various markers of neuronal injury, synaptic dysfunction, neuroinflammation, and vascular dysregulation. The latter may be important in highlighting other biochemical aspects of AD and may prove useful in late-onset AD, whether or not heterogeneous mechanisms and additional pathologies are present. This review is highly educational and has become “Editor’s choice”.

In the original article by Bourbouli et al. [3], data on CSF and plasma biomarkers along with genotypic profiling were presented in a cohort of 130 patients with frontotemporal dementia and/or amyotrophic lateral sclerosis (FTD-ALS spectrum). Patients with *C9orf72* repeat expansion, or causative variants in other genes such as *TARDBP*, *GRN*, *VCP*, and *FUS* were identified. The authors observed that some patients with *C9orf72* repeat expansions may present with lower CSF levels of τ_P-181_, while the presence of rare *C9orf72* or *APP* variants may be associated with lower levels of τ_T_ or Aβ_42_, respectively. These possible associations between genotype/phenotype and CSF biomarker levels may prove useful in the FTD-ALS spectrum, but further research is needed.

Ntymenou et al. [4] reviewed the role of plasma biomarkers in the differential diagnosis of FTD. Two biomarkers may be of particular importance. Plasma progranulin levels are reduced in the presence of *GRN* mutations, and thus, this biomarker may serve as a screening tool, identifying subjects suitable for appropriate genetic testing in FTD. On the other hand, plasma levels of phospho-tau may prove useful in the discrimination between AD and FTD, since phospho-tau seems to be normal in FTD, but increased in AD.

Katayama et al. [5] reviewed the role of CSF biomarkers in Parkinson’s disease (PD). Various biomarkers have been studied, including the classical AD biomarkers, α-syn, NFL, markers of inflammation, markers of oxidative stress, and growth factors. The authors reached four conclusions: (a) α-syn is decreased in PD, (b) decreased Aβ_42_ is a marker of cognitive decline in PD, (c) increased levels of τ_T_, τ_P-181_ and NFL are useful in differentiating PD from other related neurodegenerative disorders, and (d) some inflammation-related markers such as IL-1β, IL-6, and TGF-β are increased in PD. These conclusions are widely accepted among experts in Parkinsonism, and this paper has become “Editor’s choice”.

Although reduced α-syn may be considered a marker of PD [5], results concerning α-syn levels in other related movement and cognitive disorders are conflicting, probably due to methodological differences among the various studies, which may measure different forms of α-synuclein. Thus, currently, α-syn is considered as an emerging, but not as an established biomarker. Constantinides et al. [6] performed an interesting study in a total of 135 patients including PD, multiple system atrophy (MSA), progressive supranuclear palsy (PSP), corticobasal degeneration (CBD), vascular dementia, FTD, and AD. They measured CSF levels of various α-syn species, such as total α-syn (t-α-syn), phosphorylated α-syn (pS129-α-syn) and α-syn oligomers (o-α-syn). They observed that t-α-syn was lower and the t-α-syn/pS129-α-syn ratio was higher in synucleinopathies (PD and MSA), as compared to tauopathies (PSP and CBD), providing new data on the possible role of different α-syn species in the biochemical mechanisms and the differential diagnosis of movement disorders.

Biomarkers may lead to a correction of the antemortem diagnosis of neurodegenerative diseases. This is useful in everyday practice for applying the appropriate, currently approved treatments (or avoiding contraindicated ones). Since disease-modifying treatments for AD are currently tested and starting to be approved (including anti-Aβ antibodies), correct diagnosis may be more important than ever, for correct recruitment in clinical trials, for applying the new drugs in early or clinically atypical cases of AD, and for avoiding their use in “amnestic-like” but non-AD cases. In this context, Paraskevas and Kapaki [7] presented a short review and, according to the currently accepted concept of AD [1], they suggested that Alzheimer’s disease should be diagnosed when both Aβ_42_ and τ_P-181_ are abnormal. The reduction of Aβ_42_ alone may not be sufficient, despite the fact that sometimes it may be indicative of the Alzheimer’s continuum.

Interestingly enough, Endres et al. [8] reported a female patient with a frontal psychiatric–behavioral and cognitive presentation, combined with a Parkinsonian syndrome. The clinical picture could be compatible with a 4-repeat tauopathy, such as frontal-type PSP. However, anti-glycine receptor antibodies (anti-GlyR) were found. The authors hypothesized that either the anti-GlyR disease presented with an atypical phenotype, or the neurodegenerative disorder and the autoantibody production, were coincidental and unrelated. However, they also suggested a more tempting scenario: the neurodegenerative disorder was followed by secondary autoantibody production, suggestive of an interplay between neurodegeneration and neuroinflammation. Whatever the explanation, this case report reminds us that secondary causes should not be missed, especially in cases with atypical presentations.

The articles published in this Special Issue contribute to the advancement of our understanding of biochemical markers in dementing disorders, including the neurodegenerative proteinopathies.
